# Borderline Personality Disorder With Atypical Traits in a 30-Year-Old Female: A Case Report

**DOI:** 10.7759/cureus.55166

**Published:** 2024-02-28

**Authors:** Sarah Garikana, Pratik Jain, James L Megna, Luba Leontieva

**Affiliations:** 1 Psychiatry, State University of New York Upstate Medical University, Syracuse, USA; 2 Psychiatry and Behavioral Sciences, State University of New York Upstate Medical University, Syracuse, USA

**Keywords:** borderline personality disorder subtypes, occupational functioning, biopsychosocial model, atypical presentations, high-functioning internalizing, psychological defense mechanism, borderline personality disorder

## Abstract

In this paper, we report an atypical presentation of borderline personality disorder (BPD) in a 30-year-old female with a history of childhood molestation and trauma and a prior diagnosis of post-traumatic stress disorder (PTSD). The patient was hospitalized due to anxiety, depression, and guilt over her relapse into alcohol use disorder. During her hospital stay, we diagnosed her with BPD based on psychiatric examination, clinical interviews, and patient history. While the patient exhibited some of the typical characteristics of BPD, such as an instability of interpersonal and romantic relationships, there were numerous findings that were atypical of BPD. These include a demonstration of mature defense mechanisms such as sublimation and altruism, high levels of occupational functioning, strong maternal caregiving behavior, and no history of self-harm. Further analysis of the patient’s personality traits helped us identify that this presentation could be best characterized as a high-functioning internalizing subtype of BPD as identified in prior literature.

## Introduction

Borderline personality disorder (BPD) is characterized by a pervasive pattern of instability of interpersonal relationships, self-image, and affects. BPD is one of the most common personality disorders with nearly 10% prevalence among psychiatric outpatients and 15%-25% prevalence among psychiatric inpatients [1]. According to the Diagnostic and Statistical Manual of Mental Disorders, Fifth Edition (DSM-5) [2], a patient can be diagnosed with BPD if they exhibit at least five out of the nine diagnostic criteria. Therefore, there are 256 different combinations of the symptoms that can result in a BPD diagnosis [3]; thus, any two individuals with BPD can have substantially different symptoms. Additionally, there are many other mental disorders that have high comorbidity with BPD; this further adds to the diagnostic challenges [4] and diversity in the presentation of BPD. While there is a conceptual coherence about BPD, there is a large variation of symptoms observed in clinical practice [5].

In this paper, we report an atypical presentation of BPD in a 30-year-old female patient. As with most BPD patients, her romantic relationships were prone to dysfunction, volatility, and instability [6]. However, we observed numerous traits in this patient that are uncommon in BPD (e.g., mature defense mechanisms). In fact, some of the traits observed in this patient could have eliminated BPD from the differential diagnosis. Further analysis with respect to prior literature on BPD subtypes [7] helped identify that this case most closely resembles the “high-functioning internalizing” subtype [8]. This case demonstrates some of the diagnostic challenges of BPD in clinical practice due to the diversity of symptoms and presentation. Recent clinical research has shown that treatment responses, trajectories of change, and outcomes can differ based on the subtype of BPD [9,10]. We therefore expect this case to be of interest to the psychiatric community.

## Case presentation

The patient is a 30-year-old Caucasian female with a past psychiatric history of depression, anxiety, alcohol use disorder, substance use disorder, and post-traumatic stress disorder (PTSD). She has no other significant medical history, prior hospitalizations, or prior diagnosis of BPD. She came voluntarily to the hospital due to worsening depression, anxiety, and guilt because she started relapsing into alcohol use disorder. She maintained sobriety from alcohol for almost the last five years. However, over the last six months, she has been going through a custody battle with the father of her second child. She experienced three relapses during this period, each involving a few drinks over the course of a day or two. She felt that the alcohol would help alleviate the anxiety from the custody battle. However, it only triggered severe guilt, shame, and depression because she felt she had undone all the progress toward sobriety.

The patient grew up in a rural environment in a lower-income family. Both her biological parents have significant psychiatric histories. Her biological father was diagnosed with bipolar I disorder, depression, and anxiety. Her biological mother suffers from alcohol use disorder, substance use disorder (opioids), and depression. Her parents divorced when she was six years old, and they both remarried. Her maternal grandmother had depression, and her maternal uncle had schizophrenia. She also reported that other members of her family have been diagnosed with depression and schizophrenia.

The patient has a close relationship with her biological father. However, when she was younger, she often did not fully understand some of her father’s behavior, especially during his episodes of mania. One such incident was when her father, who was going through mania, started a family gardening project and spent hundreds of dollars on various gardening tools. She was very excited about this project and at the prospect of spending time with her father. However, as her father’s mania came to an end, he completely abandoned the project leaving her confused and disappointed. These types of incidents were a recurring pattern in her childhood, and they inflicted considerable emotional pain and disappointment upon her. But, as she grew up, she came to understand that many of her father’s actions and decisions were more indicative of his mental health than any deliberate disregard for her. She described the relationship with her biological mother as strained. During her childhood, her mother often called her an “angry baby,” referring to the instances of anxiety and aggression exhibited by the patient even in response to minor triggers.

Her elder sister has a history of high-risk behavior including running away from home and driving faster than speed limits. During one such driving incident, when the patient was 12 years old, her sister met with a motor vehicle accident resulting in multiple surgeries. Subsequently, the patient helped her mother take care of her sister at home during recovery. During this time, she was actively repressing her emotions to not cause any further trouble at home; she described herself during this period as “imploding.”

She was sexually molested at the age of 10 by her stepbrother, who was then 18 years old. Prior to this incident, she had a close relationship with her stepbrother, but after that, he started to distance himself and was also rude to her. These developments left her confused, and she felt abandoned. The incident did not involve any use of physical force, but the patient was below the age of consent. She did not disclose this incident to any adults at that time. She described that the incident made her feel like she was a grown-up. After this, she started having many short-term sexual relationships with strangers as a way “to let go of the anger.”

The patient first started using opioids in her early teen years. She describes her first drug use as attempts to escape from stressors at home life. She also started using alcohol and cigarettes during her high school years. She has been clean of opioids since 2011 and has been on suboxone until 2017.

The patient discussed a significant incident of trauma at the age of 15. She said that her then-best friend often fabricated stories about sexual assaults to gain sympathy from peers. She did not like what her friend was doing and told everyone that her friend was lying about these assaults. In revenge, her friend took her to a park where she arranged for a group of girls to bully and physically assault her. Her friend was watching the entire incident and later proposed a reconciliation, stating, “Now that I’ve taken revenge, we can be best friends again.” She felt extremely betrayed by her friend because of this incident and cut all ties with her. She also stated that this episode has significantly impacted her ability to form close friendships throughout her adult life.

The patient suffers from an unhealthy body image. She wore big, baggy clothes during her teenage years because she thought she was overweight, though her weight was typical for her height and age. She also has a history of disordered eating. She restricted herself to a diet of only chicken and almonds for a year, leading to a weight loss of nearly 30 pounds. Subsequently, she had bouts of binge eating causing her to regain the weight. This is an example of her impulsivity, one of the DMS-5 diagnostic criteria for BPD. At the time of admission, she described herself as overweight despite her body mass index falling within the normal range. She has no history of suicidal ideation or non-suicidal self-injurious behaviors (NSSIB).

She describes her menstruation as regular but reports increased anxiety and craving for junk food a week before the onset, which continues two weeks thereafter. During this time, she describes her mood to be “very low” and has decreased energy, increased anxiety, and insomnia.

During her adulthood, she had seven significant romantic relationships of varying lengths. She described that all the relationships start with intense infatuation for her partners, followed by periods of devaluation when she becomes more irritable. She describes herself as being “emotionally abusive” to her partners by being non-committal in relationships. When she perceives any threat of abandonment from her partner, she has intense emotional dysregulation and anxiety, leading her to actively pursue a breakup with the partner. This behavior highlights control issues observed in an abused person.

She has two children aged six and 12 from two different relationships. She met the fathers of both her children in Alcoholics Anonymous (AA) meetings. At the age of 18, she had her first child from a short-term relationship. Despite her wanting to do things a typical 18-year-old would, her mother insisted on her taking responsibility for her son. She was struggling with alcoholism during this time. Her mother gave her an ultimatum saying she either attends AA meetings or stays home to take care of her son. She chose to go to AA meetings since she did not like staying at home and taking care of her son. Over the next year, she became sober and took full responsibility for her son.

Her most significant romantic relationship is with the father of her second child, with whom she was in a five-year relationship and was also engaged. She described him as “kind and loving”; he supported her through episodes of depression and anxiety. Two months prior to their planned wedding, she started experiencing self-doubt and anxiety. Driven by fear of impending changes in her life, she cheated on him, leading to a relationship dissolution. Since this incident, she reports worsening anxiety attacks, guilt, and depression.

At the time of her admission, she was involved in a six-month relationship. When her partner suggested to move in with him, she swiftly agreed, but a few days later, she had doubts and anxiety about the decision, leading her to return home. A few days later, she started struggling with guilt and anxiety over her decision and concerns about her partner’s perception and eventually moved back in with him. She describes herself as “fickle minded,” and she struggles with inconsistency in her feelings and decisions.

Hospital course

During her hospitalization of two weeks, she attended dialectical behavior therapy (DBT) groups and was started on sertraline 50 mg for her depression and anxiety. She was also given naltrexone 50 mg to help with her alcohol use disorder. Table 1 shows various tests administered to the patient and their corresponding findings.

**Table 1:** Psychiatric tests administered to the patient and the corresponding outcomes PTSD, post-traumatic stress disorder; DSM-5, Diagnostic and Statistical Manual of Mental Disorders, Fifth Edition

Test	Scores
Outcome Questionnaire (OQ-45.2)	Overall score: 73
OQ-45.2 Symptom Distress (SD)	Score: 45
OQ-45.2 Interpersonal Relations (IR)	Score: 23
OQ-45.2 Social Role (SR)	Score: 5
General Anxiety Disorder-7 (GAD-7)	Score: 16
PTSD Checklist for DSM-5 (PCL-5)	Score: 50
Beck Depression Inventory (BDI-II)	Score: 17

The results from her OQ-45.2 [11] suggest an overall level of moderate distress, mainly through symptom distress and interpersonal relationships, but lower levels of distress in her social role. Her PCL-5 scores are well above the cutoff of 33, which is suggestive of PTSD [12]. Her General Anxiety Disorder-7 (GAD-7) and Beck Depression Inventory II (BDI-II) results show severe levels of anxiety and moderate levels of depression, respectively. We also conducted Structured Clinical Interview for DSM-5 (SCID); the results are shown in Table 2. She got a score of 3 for seven out of the nine diagnostic criteria, thus helping with our BPD diagnosis.

**Table 2:** Results of SCID SCID, Structured Clinical Interview for DSM-5; DSM-5, Diagnostic and Statistical Manual of Mental Disorders, Fifth Edition

Diagnostic criteria	Score
Frantic efforts to avoid real or imagined abandonment	3
Pattern of unstable and intense interpersonal relationships	3
Identity disturbance	3
Impulsivity	3
Recurrent suicidal behavior	1
Affect instability	3
Chronic feeling of emptiness	3
Inappropriate, intense anger	2
Transient, stress-related dissociative symptoms	3

## Discussion

When we examined the patient’s romantic relationships, one of the common patterns we observed is that when faced with any prospect of abandonment (e.g., an argument with her partner or her partner leaving home for a day), she would act out becoming anxious, aggressive, panicky, and sometimes verbally abusive. She would also become emotionally abusive to her partners by being non-committal in her relationships. She would then take action toward a dissolution of her romantic relationship to avoid any anxiety or discomfort that might stem from her partner breaking up with her. The patient associated this repetitive pattern with her history of childhood molestation by her stepbrother and the traumatic incident involving betrayal from her best friend. In both these incidents, she felt abandoned by individuals with whom she had very close interpersonal relationships. These incidents contributed to the patterns of distancing herself from intimate partners. The patient’s management of romantic relationships illustrates that her acting-out defense mechanism is quite prominent. This is one trait we observed in her that is typical of BPD patients: the display of immature acting-out defense mechanism [13].

Atypical findings

The patient has no prior history of hospitalization; she visited psychiatric clinics for her issues with alcoholism and smoking where she was diagnosed with PTSD. Despite the patient displaying instability in her interpersonal relationships, she was never previously diagnosed with BPD. This is likely because she has no history of suicidal behavior or NSSIB. Suicide attempts and self-injurious behaviors are highly prevalent in patients with BPD; prior research shows 90% rates of self-mutilation in both adult and adolescent BPD patients [14].

The patient has two children and has a very close relationship with both. She mentioned that one of her main motivations behind admitting herself to the hospital was so that she could recover from her alcohol use disorder and take care of her kids. She has demonstrated very strong maternal caregiving behavior, which is atypical of mothers with BPD, who are strongly associated with maladaptive parenting and maternal emotional dysfunction [15]. This also demonstrates a mature defense mechanism in the patient, specifically altruism, where she is dealing with emotional conflicts by dedicating herself to fulfilling the needs of others.

The patient has a successful employment history for almost eight years demonstrating high levels of occupational functioning. This is again an atypical observation since prior research has shown that people with BPD tend to have low levels of occupational functioning [16].

She has also maintained nearly 12 years of sobriety from opioids and five years of alcohol sobriety. These are signs of a mature sublimation defense where she is channeling strong emotions and feelings into positive and socially acceptable behavior.

High-functioning subtype

As discussed earlier, the BPD population is very heterogenous with a wide variance in presentation contributing to the diagnostic challenges in clinical setups. To address these issues, researchers have been working on identifying distinct subtypes of BPD and detecting homogenous groups of BPD patients with similar personality traits or symptoms using various statistical approaches. Most research to date has suggested the existence of three to four subtypes of BPD [7]. One of the more widely used categorizations was developed by Bradley et al. [8], where the authors applied Q-factor analysis on BPD patients’ data containing personality descriptors identified by clinicians. The authors discovered four naturally occurring subtypes of BPD labelled as high-functioning internalizing, histrionic, depressive internalizing, and angry externalizing. While these subtypes have some common characteristics (e.g., unstable self-image and emotional dysregulation), there are several distinct properties that characterize each subtype.

Bradley et al. [8] have identified 13 personality traits, where the high-functioning internalizing subtype scores significantly higher than the others. We observed at least eight out of the 13 personality traits in our patient. These include her tendency to feel anxious, empty, guilty, and false or fraudulent. Her feelings of guilt were most evident when she relapsed into alcohol use. We also observed that she has good psychological insight, another one of the personality traits, specifically insight into distress and anxiety from a breakup. She was also self-critical, displayed self-blame (feels responsible for bad things that happen), and oscillated between under control and overcontrol of needs and impulses. Additionally, the patients of this subtype are less likely to form friendships, become physically aggressive, or be outgoing; traits we observed in our patient. There were a few characteristics of this subtype that we did not explicitly observe; these include the tendency to elicit liking in others, moral and ethical standards, conscientiousness, and creativity. This type of BPD subtype analysis could be important in clinical setups: it can help with appropriately targeted intervention since treatment responses and outcomes can differ based on the BPD subtype [9,10].

Biopsychosocial

We employed the biopsychosocial model to further understand the patient’s psychopathology by considering biological, psychological, and social factors impacting her BPD. The questionnaire used for this exercise was developed by the psychiatric inpatient unit at the State University of New York (SUNY) Upstate.

Biologically, for her BPD, she is vulnerable to depression given her family’s history of mental illnesses. We expect that her history of childhood abuse and trauma could be causative factors [17] for her BPD. Prior research shows that childhood abuse can impact long-term brain development, often leading to hippocampal volume reduction [18]. Her familial history of alcoholism was a risk factor in her adolescent alcohol initiation [19]. Additionally, the comorbidity of BPD and alcohol use has been documented in prior research [20]. Though none of her direct family members were diagnosed with BPD, we observed that her elder sister displayed a few BPD traits. Familial and twin studies support the role of genetics in BPD, with heritability estimated to be approximately 40% [21]. Environment factors such as parental conflict, as is the case with our patient, have also been strong predictors of offspring BPD traits [22]. The severity of her premenstrual syndrome (PMS) and premenstrual dysphoric disorder (PMDD) could also be related to her personality disorders [23]. We expect that treating her PMS and PMDD would be beneficial to her overall mental health.

From a psychological perspective, we observed that her level of personality disorganization is borderline, and her ego strength is poor. Her immature defense mechanisms include acting out, splitting, idealization, and devaluation. She also displayed a neurotic level of dissociation. Her mature defense mechanisms include sublimation and altruism. She has poor impulse control, labile mood, fair judgement, poor anxiety tolerance, and poor relational capacity but good work capacity. We expect that DBT in both individual and group settings would benefit her.

Socially, the patient’s pattern of relatedness as an adult is characterized by abusive, explosive, unstable, and intense affect. Her ability to form and maintain long-term friendships was significantly marred due to the incident of trauma at the age of 15. We expect that if she develops more friendships, it can help with her overall mental health. We also recommend depression support groups and psychosocial clubs for forming supportive relationships.

## Conclusions

The BPD population is very heterogenous; it is very important to present unusual cases to the psychiatric community as it can shed light on some of the diagnostic challenges and potential treatment options. In this paper, we present an atypical presentation of BPD in a 30-year-old female, where the patient demonstrated characteristics such as mature defenses, maternal caregiving, and high occupational functioning. The patient went undiagnosed for many years likely because she never exhibited any patterns of self-harm. We analyzed the patient with respect to BPD subtypes, and she can be best characterized as high-functioning internalizing.
